# Glucose Sensing Using Capacitive Biosensor Based on Polyvinylidene Fluoride Thin Film

**DOI:** 10.3390/bios8010012

**Published:** 2018-01-30

**Authors:** Ambran Hartono, Edi Sanjaya, Ramli Ramli

**Affiliations:** 1 Department of Physics, Faculty of Sciences and Technology, UIN Syarif Hidayatullah Jakarta, Tangerang Selatan, Banten 15412, Indonesia; ambran.hartono@uinjkt.ac.id (A.H.); edi.sanjaya@uinjkt.ac.id (E.S.); 2 Department of Physics, Faculty of Mathematics and Natural Sciences, Universitas Negeri Padang, Padang 25131, Indonesia

**Keywords:** capacitive biosensor, glucose sensing, GOx enzyme, PVDF film

## Abstract

A polyvinylidene fluoride (PVDF) film-based capacitive biosensor was developed for glucose sensing. This device consists of a PVDF film sandwiched between two electrodes. A capacitive biosensor measures the dielectric properties of the dielectric layers at the interface between the electrolyte and the electrode. A glucose oxidase (GOx) enzyme was immobilized onto the electrode to oxidize glucose. In practice, the biochemical reaction of glucose with the GOx enzyme generates free electron carriers. Consequently, the potential difference between the electrodes is increased, resulting in a measurable voltage output of the biosensor. The device was tested for various glucose concentrations in the range of 0.013 to 5.85 M, and various GOx enzyme concentrations between 4882.8 and 2.5 million units/L. We found that the sensor output increased with increasing glucose concentration up to 5.85 M. These results indicate that the PVDF film-based capacitive biosensors can be properly applied to glucose sensing and provide opportunities for the low-cost fabrication of glucose-based biosensors based on PVDF materials.

## 1. Introduction

According to a World Health Organization (WHO) report on diabetes in 2016 [1], diabetes is a chronic and progressive disease characterized by elevated blood glucose levels. Diabetes is one of the leading causes of death and disability, such as blindness, nerve degeneration, and kidney failure [2]. Therefore, early monitoring of blood glucose levels is crucial. Developments in glucose sensing for diabetes monitoring has been reported [3]. The first generation of glucose biosensors was reported by Clark and Lyons in 1962 [4]. Various technologies have been used for glucose sensing. Among them are infrared spectroscopy [5], fluorescence spectroscopy [6], Raman spectroscopy [7], liquid chromatography [8], polarimetry [9], and impedance spectroscopy [10]. However, expensive equipment requirements and complicated operations have become some of the obstacles to accessible glucose monitoring.

A promising tool for sensing glucose is a capacitive biosensor. Capacitive biosensors are electronic sensors that work based on the concept of capacitive sensing. The sensor works based on changes in the electrical energy charge that can be stored by the sensor as a result of changes in the distance plate, changes in the cross-sectional area, and volume changes in the dielectric of the capacitive material [11]. The capacitive biosensors measure the changes in dielectric properties at the interface between the electrode and electrolyte when the analyte interacts with the immobilized receptors on the isolated dielectric layer [12]. The use of capacitive biosensors in glucose detection has been reported by several researchers [13,14,15,16,17]. 

**Figure 1:**
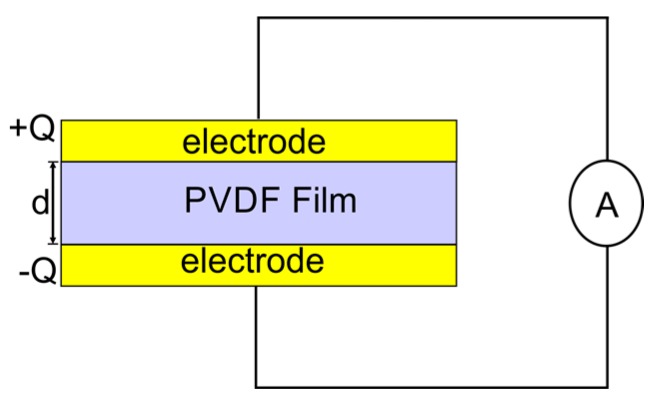
Schematic diagram of a capacitive sensor based on polyvinylidene fluoride (PVDF) film.

The construction of a capacitive sensor involves a polyvinylidene fluoride (PVDF) film sandwiched between two electrodes, as shown in Figure 1. If a voltage difference exists between the two electrodes, a capacitance between them will appear. The capacitance produced is proportional to the surface area of the electrodes and the dielectric constant, and is inversely proportional to the distance between the two electrodes. Different applications of capacitive biosensors developed for different targets have been reported [11].

In this paper, we present the concept of capacitive sensors for glucose sensing. The detection of glucose works through the enzymatic catalysis reaction that produces or consumes electrons. The enzymes are appropriately called redox enzymes [18]. The sensor substrate usually consists of three electrodes: the reference electrode, the working electrode, and the counter electrode. Target analytes are involved in the reactions that occur on the surface of the active electrode, and the reaction can lead to the transfer of electrons in the dielectric layer or can contribute to the layer. The measured flow rate is proportional to the flow of electrons in the dielectric and is equivalent to the analyte concentration [19].

**Figure 2:**
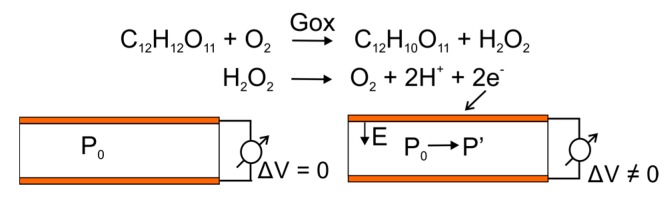
The schematic capacitive sensor before and after the reaction.

In principle, the detection of glucose works as a capacitive sensor where the sensor component consists of two electrodes (silver metal) sandwiching a dielectric material (PVDF film). Both electrodes are connected to the I–V meter, as shown in Figure 2. The initial state before the reaction of measuring instruments showed no signal. At the first dielectric material, PVDF has a polarity P0, and the potential difference between the electrodes ΔV is 0. The chemical reaction between glucose and the free air with the GOx enzyme catalyst generates free charge carriers (electrons). The concentration of the charge on the electrodes causes the polarity of the electric field that affects the dielectric material.

The polarity of dielectric P_0_ transforms into P′. This polarity change, ΔP = P_0_ − P′, causes the potential difference of the electrodes (ΔV ≠ 0). Empirically, the amount of current detected is dependent on the potential difference. The potential difference generated depends on the amount of chemical reagent concentration, for both glucose and enzymes. In this case, the concentration charge of the chemical reaction results cause the flow of electric charges that are detected in the form of voltage (V). In other words, the magnitude of the output voltage is proportional to the amount of charge generated from the chemical reaction [20].

## 2. Materials and Methods

### 2.1. Fabrication of PVDF Thin Film as a Capacitive Sensor

In this research, the PVDF film was fabricated using the deep coating method. The deep coating technique is a technique used to make a thin polymer film with the dissolution method. The PVDF powder is dissolved in a dimethyl formamide (DMF) solvent, and the film is produced through a PVDF solution coating process on the glass substrate by using a deep coating machine. 

The initial stage of this experiment involved the manufacturing of PVDF thin films by making PVDF solutions with various concentrations. PVDF powder was dissolved in DMF solvent. The PVDF solution was made for three different concentrations, 10%, 15%, and 20%, referred to as S#1, S#2, and S#3. The dissolution of PVDF powder was conducted in a beaker glass and stirred using a magnetic stirrer. The dissolution process was performed for 60 min, and a temperature of 100 °C was maintained to obtain a homogeneous solution.

The fabrication of PVDF thin films was first performed for the PVDF solution with a concentration of 10%. Dyeing was completed once and the duration of immersion was 30 h. Next, the PVDF film was dried and then exfoliated from glass preparations. Thereafter, the thin PVDF films were annealed for temperature variations of 70 °C, 80 °C, 90 °C, 100 °C, and 110 °C. Samples of thin PVDF films were characterized by Fourier Transform Infrared Spectroscopy (FTIR) and X-Ray Diffraction (XRD).

**Figure 3:**
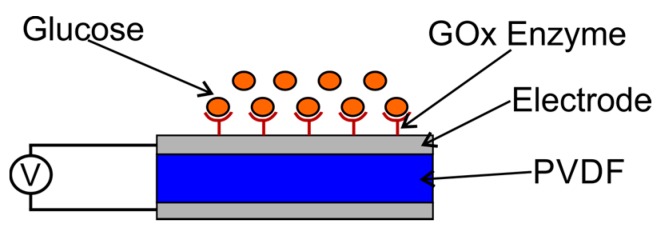
The scheme for glucose detection with a capacitive sensor [21].

PVDF film that was produced and had good piezoelectric quality was used as a sensor element, then coated with silver as an electrode, which was then assembled into a sensor instrument. The sensor design is shown in Figure 3. 

### 2.2. Glucose Sensing

The experimental stages involved preparing materials and equipment, making the GOx enzyme and glucose solutions, coating the GOx enzyme, and sensor testing. The materials used in this experiment were GOx enzyme ((Sigma Aldrich, Singapore), saccharine (Sigma Aldrich, Singapore), 70% alcohol (Sigma Aldrich, Singapore), and Aqua Dest (Merck, Indonesia). The equipment used included dropper drops (0.5 mL, 1 mL, and 5 mL), sample bottle, measuring cup, beaker glass, sample box, PVDF film, and I–V meter. 

#### 2.2.1. Preparation of GOx Enzyme Solution

The steps for making the solution involved 25 KU (Kilo unit, 1 KU = 100 mg) of GOx enzyme dissolved in 10 mL Aqua Dest (25 KU/10 mL), called Z1 solution. The Z1 solution was then divided into two parts of 5 mL each. One part of the Z1 solution was dissolved in 5 mL of Aqua Dest (25 KU/20 mL), called Z2 solution. Z2 solution was again divided into two parts of 5 mL each. One part of the Z2 solution was then dissolved in 5 mL Aqua Dest to obtain Z3 (25 KU/40 mL). The same process was completed until a GOx enzyme solution was obtained with 10 concentrations, as shown in Table 1.

The GOx enzyme acts as a bio-receptor in the sensor design. Therefore, the PVDF films should be integrated with enzymes, with a coating of GOx enzyme solution on the PVDF film surface. The coated film was allowed to sit for 1 h until the enzyme solution dried. This step was repeated for all enzyme solutions with different concentrations that had been made.

#### 2.2.2. Preparation of Glucose Solution

The glucose solution to be measured by the sensor was made with various concentrations, as shown in Table 2. 

#### 2.2.3. Sensor Measurement

Sensory testing was performed by dripping a glucose solution over a PVDF film coated with the GOx enzyme. The biochemical reaction will occur, resulting in free charge carriers (electrons). A number of free electrons in the PVDF films result in the emergence of a field affecting the polarity of the PVDF films. Consequently, this field causes electrical displacement and produces a potential difference that can be read on I–V meters. The amount of potential read on the I–V meter is influenced by the amount of reactant concentration, including glucose solution and enzyme concentration. 

## 3. Results and Discussion

### Glucose Sensing 

Based on the results of FTIR and XRD characterization, as shown Figures S1 and S2, the PVDF film used as a glucose sensor was sample S#5. The theory that biochemical reactions in the sensors are strongly influenced by the concentration of the catalyst and reactants was demonstrated by the curve of the potential measurement results from given enzyme and glucose concentration conditions. Next, we compared the potential versus time curve for various enzyme and glucose concentrations.

**Figure 4:**
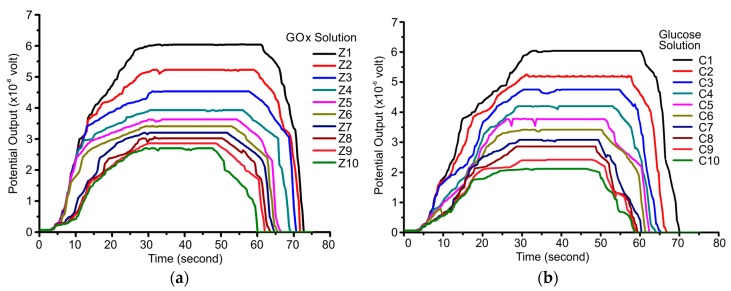
Potential versus time for (**a**) various enzyme concentrations with a glucose concentration of 5.85 M, and (**b**) various glucose concentrations with an enzyme concentration of 2,500,000 units/L.

Figure 4a shows the potential versus time curve of the sensor for various enzyme concentrations with a glucose concentration of 5.85 M. Figure 4b shows the potential versus time curve of the sensor for various glucose concentrations with a GOx concentration of 2,500,000 units/L. 

According to Figure 4a,b, the resulting curves have an almost identical shape. The potential increases, followed by a constant potential moment at a certain value. These parameters will be reviewed for further analysis as they relate to the reaction rate and enzyme activity of the biochemical reactions occurring. The relationship between potential and time shows a relationship of exponential equations up to a certain time. This potential shows the dynamics of the enzymatic reaction between the GOx enzymes and glucose. The reaction between GOx enzymes and glucose will produce electrons. In an oxidized state, the enzyme and glucose reactions produce gluconic acid and release two electrons and two protons. The electron of this reaction changes the concentration of the charge on the PVDF film as a dielectric material.

The number of these electrons depends on the amount of hydrogen peroxide. The amount of hydrogen peroxide depends on the glucose concentration. Consequently, the higher the concentration of glucose, the more electrons are generated. This is shown by the amount of potential produced for various glucose concentrations. 

**Figure 5:**
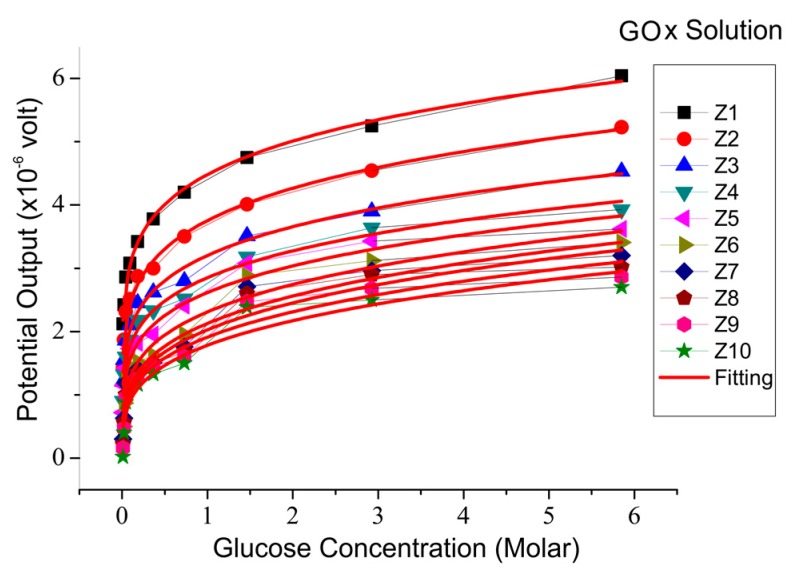
Sensor output voltage compared to glucose concentrations for various enzyme concentrations.

**Figure 6:**
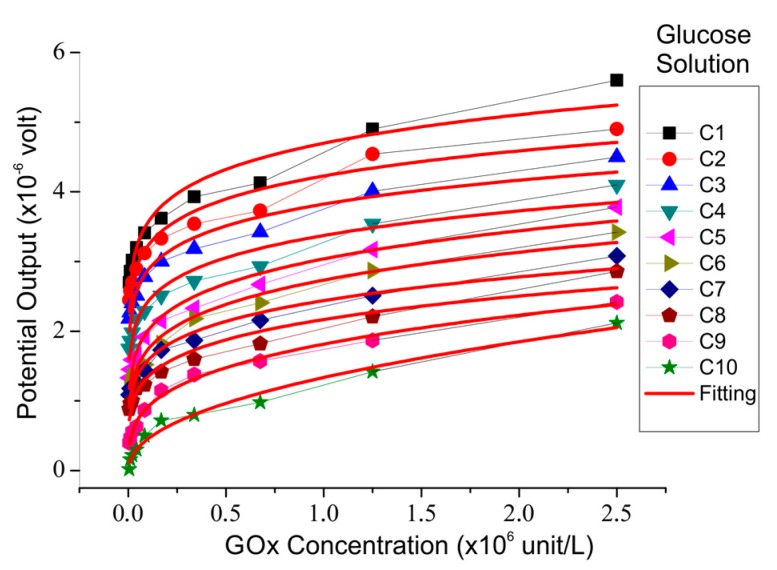
Sensor output voltage compared to enzyme concentrations for various glucose concentrations.

The relationship between the sensor output potential and the glucose concentration is shown in Figure 5. The sensor output increased with increasing glucose concentration up to 5.85 M. In theory, the rate of enzyme reaction will increase with the concentration of the reacting substrate. Figure 5 is analogous to the saturation curve of the enzyme reactions, as shown in Figure 6, in which the parameter that states the rate of reaction is the potential. The rate of reaction states the number of reactions that occur with every unit of time. In this case, time is expressed by the concentration of solute in the reaction produced every second of the reaction.

Electrochemical biosensors are usually based on enzymatic catalysis reactions that produce or consume electrons. The enzyme involved is aptly called the redox enzyme. Substrate sensors usually contain three electrodes: reference electrode, working electrode, and counter electrode. The target analyte is involved in the reaction occurring on the surface of the active electrode, and the reaction may cause electron transfer in the dielectric layer, producing current, or it may contribute to the coating, producing voltage. The measured current is proportional to the electron flow rate in the dielectric equivalent to the concentration of the analyte.

In the experiment, the substrate in Figure 6 is glucose. The relationship between the potential and the rate of reaction is seen from the current definition, i.e., the amount of charge flowing per time, in units of Coulomb/second. Therefore, the current represents the reaction rate. The more electrical charge that flows into the dielectric material, the greater the rate of reaction. The reaction rate of the biochemical reaction between the GOx enzyme and glucose (*v*) is a function of potential (*V*), and can be expressed by Equation (1):

*v* = *τ V* (1)

where *τ* is a constant.

The reaction rate per mol is obtained from the generated saturation potential value. Based on the experimental results, the increase in saturation potential resembles the exponential equation and is expressed by Equation (2).

(2) $$V=V_{0}\cdot e^{R_{0}\cdot x}$$

where *V*_0_ is the initial potential, and *A* and *R*_0_ are constants. 

The second part of the right-hand side of Equation (2) requires further study because it involves various parameters affecting the rate of biochemical reactions as described by the Arrhenius equation. In this paper, we examine the parameters associated with reaction rates due to changes in glucose and enzyme concentrations.

The reaction rate per mole was obtained based on the slope of the graph in Figure 6. Based on the result of the graph curve, the value of each constant could be obtained by matching the value of the sensor output to the glucose concentration for various enzyme concentrations, as listed in Table 3. 

Furthermore, the effect of the reactant concentration on the polarization change from the PVDF film will be presented. To analyze its influence, we began by reviewing experimental results and theoretical studies. Based on the experimental data and theoretical study of the working principle of capacitive sensors, the values of stress and charge for various concentrations of glucose at fixed enzyme concentrations are 2,500,000 units/L, as shown in Table 4. 

Table 4 shows that the greater the glucose concentration, the greater the potential produced. Theoretically, this can be understood as the greater the concentration of glucose, the greater the charge released from the reaction, so the greater the potential difference.

**Figure 7:**
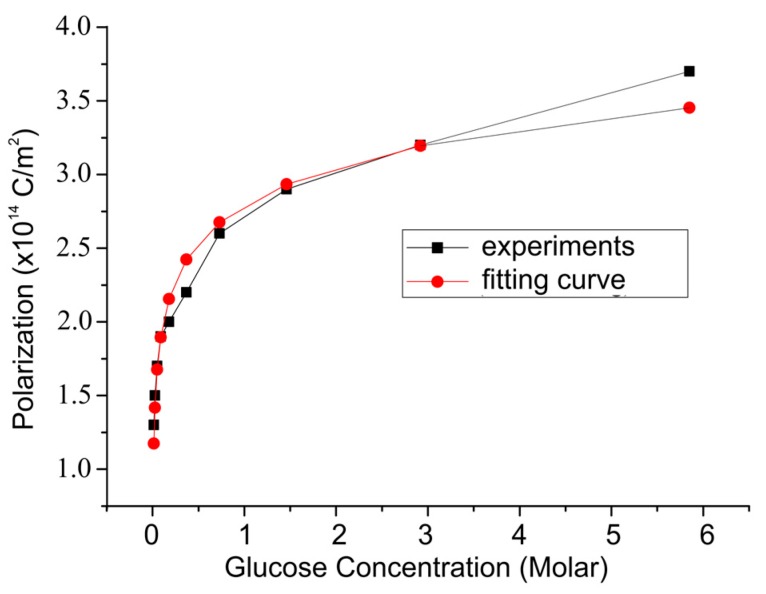
The change in PVDF film polarization versus glucose concentration with an enzyme concentration of 2,500,000 units/L.

According to Table 4, the value of the electric field (*E*) arising from various glucose concentrations can be calculated, and the number of dipole moments can be determined. Those quantities were used to obtain the polarization changes in PVDF films, as shown in Figure 7.

The capacitive biosensor based on the PVDF film for glucose detection was performed, having a dynamic range of 0.013–5.85 M and limit of detection of 0.013 M. This result is larger than the range and limit of detection of the capacitive biosensors that have been developed by other researchers, as shown in Table 5.

The presence of enzymatic reactions between GOx and glucose enzymes results in free charge carriers. This free charge creates an electric field that causes a change in the dielectric polarity of the PVDF film. Consequently, an electric movement occurs in the PVDF film. The greater the concentration of glucose, the greater the change in PVDF polarity. This phenomenon indicates that PVDF films function well as capacitive sensors. We suspect that this result is related to the phenomena called chemico-electrical mechanisms. This mechanism can be explained as follows. The electric field from the GOx enzyme and glucose reaction affects the polarization in the dipole orientation of the PVDF film. This polarization is related to changes in chemical reactions that occur between the GOx enzyme and glucose, via Equation (3).

(3) $$dP={\left(\frac{\partial P}{\partial S}\right)}_{E=0;T}dS+{\left(\frac{\partial P}{\partial T}\right)}_{E;S}\chi^{S}\varepsilon_{0}dE+\left(\frac{\partial P}{\partial C}\right)dC+\left(\frac{\partial P}{\partial Z}\right)dZ$$

where *P* is polarization, *S* is strain, *T* is temperature, *E* is the electric field, χ is susceptibility, *ε*_0_ is the vacuum dielectric permittivity, *C* is glucose concentration, and *Z* is the enzyme concentration. 

The change in polarization due to the change of chemical reactions is expressed in Equations (4) and (5).

(4) $$dP=\left(\frac{\partial P}{\partial C}\right)dC+\left(\frac{\partial P}{\partial Z}\right)dZ$$

*P* − *P_r_* = *c*(*C* − *C*_0_) + *z*(*Z* − *Z*_0_) (5)

where *c* is the reactant constant and *z* is the enzyme constant. These constants associated with the reaction rate are due to changes in the reactant concentration.

## 4. Conclusions

In summary, we discovered a glucose sensing technique using a capacitive biosensor based on a PVDF film. The sensor output potential increases with increasing concentrations of glucose and GOx enzymes. The reaction rate between the GOx enzyme and glucose increases with increasing glucose and enzyme concentrations. The concentration of enzymes and glucose influences the change in PVDF film polarization through chemico-electrical mechanisms. This event shows the relationship of the enzyme and glucose concentration parameters to the polarization, following an exponential equation. We concluded that the PVDF film-based capacitive biosensors can be properly applied to glucose sensing. The dynamics range and detection limits of the capacitive biosensors that we developed are low, but still warrant further development. The results of this study provide opportunities for the fabrication of glucose-based biosensors based on PVDF materials in the future.

## Figures and Tables

**Table 1:** Concentration of glucose oxidase (Gox) enzyme solution.

GOx Solution	Concentration (U/L)
Z1	2,500,000
Z2	1,250,000
Z3	625,000
Z4	312,500
Z5	156,250
Z6	78,125
Z7	39,062.5
Z8	19,531.25
Z9	9765.625
Z10	4882.813

**Table 2:** Concentrations of the glucose solutions.

Glucose Solution	Concentration (M)
C1	5.85
C2	2.92
C3	1.46
C4	0.73
C5	0.37
C6	0.18
C7	0.09
C8	0.05
C9	0.025
C10	0.013

**Table 3:** Value of V_0_ and R_0_ in Equation (2).

Enzyme Concentration	Constants	
(Unit/L)	V_0_ (×10^−6^ volt)	R_0_ (×10^−9^ mol/s)
2,500,000.00	6.360	−0.359
1,250,000.00	5.416	−0.430
675,000.00	4.720	−0.418
337,000.00	3.992	−0.623
168,750.00	3.607	−0.986
84,375.00	3.384	−0.942
42,187.5	3.496	−0.928
21,093.75	3.054	−0.948
10,546.88	2.869	−0.922
5273.44	2.699	−1.007

**Table 4:** The value of current, charge, and potential for various concentrations of glucose with a fixed enzyme concentration of 2,500,000 units/L.

Glucose Concentration (M)	V (×10^−6^ volt)	Q (×10^−14^ C)	I (×10^−15^ A)
5.85	6.04	4.83	1.61
2.92	5.25	4.20	1.40
1.46	4.75	3.80	1.27
0.73	4.20	3.36	1.12
0.37	3.78	3.02	1.00
0.18	3.42	2.74	0.91
0.09	3.08	2.46	0.82
0.05	2.86	2.29	0.76
0.025	2.42	1.94	0.65
0.013	2.12	1.70	0.57

**Table 5:** Dynamics range, limit of detection, and sensitivity of capacitive biosensors for glucose detection.

Sensor Method	Dynamic Range (M)	Limit of Detection (M)	Sensitivity	Ref.
Immobilization of Concanavalin A (ConA) on gold nanoparticles incorporated on the tyramine-modified gold electrode	1.0 × 10^−6^–1.0 × 10^−2^	1.0 × 10^−6^	A negligible loss in sensitivity after 10 cycles (7.5%)	[15]
Immobilization of Concanavalin A (ConA) and replacement of small glucose with the large glucose polymer	1.0 × 10^−5^–1.0 × 10^−1^	1.0 × 10^−6^	N/D	[16]
Glucose solution over a PVDF film coated with the GOx enzyme	1.3 × 10^−2^–5.85	1.3 × 10^−2^	N/D	This work

## Supplementary Materials

The following are available online at http://www.mdpi.com/2079-6374/8/1/12/s1: Figure S1. The Fourier Transform Infrared (FTIR) results for the PVDF film with different solution concentrations. Figure S2. The X-ray Diffraction (XRD) results for the PVDF film with different annealing temperatures.
