# Supplementary material for: Glucose Sensing Using Capacitive Biosensor Based on Polyvinylidene Fluoride Thin Film
Source: Biosensors (Basel). 2018 Jan 30;8(1):12. doi: 10.3390/bios8010012 (PMC5872060; doi:10.3390/bios8010012)
Supplement: Supplementary file 1 [file biosensors-08-00012-s001.pdf]

Supplementary Material: Glucose Sensing Using Capacitive Biosensor Based on Polyvinylidene Fluoride Thin Film

Ambran Hartono [1], Edi Sanjaya[1], and Ramli Ramli [2,*]

1. PVDF Thin Film Characterization

FTIR characterization has been used to confirm the β fraction of PVDF samples. The FTIR results of PVDF films for PVDF solution concentrations of 10%, 15% and 20% are shown in Figure S1. It appears that for a sample with a PVDF solution concentration of 20% it has a larger β faction compared to the other two concentrations. The *α* phase appears in wavelength regions 615, 760, 973, 1230 and 1400 cm^{-1}, γ phase is detected at 1230 cm^{-1}. Meanwhile, for β phase is appearing in 840, 880, 1075 and 1170 cm^{-1}. The β fraction of the PVDF sample with solution concentration 10 %, 15% and 20% are; 31%, 39% and 44.5%, respectively.

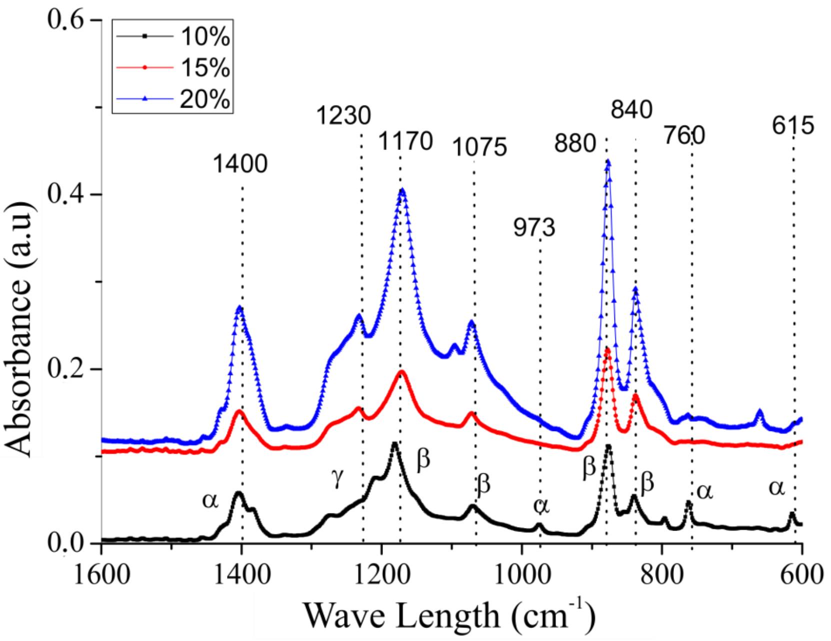

Figure S1. The FTIR results for PVDF film with different solution concentrations.

Furthermore, XRD characterization was performed on a PVDF sample with a concentration of 20% (S#3) for annealing temperature of 70°C, 80°C, 90°C, 100°C, and 110°C (referred as SS#1, SS#2, SS#3, SS#4, and SS#5), as shown in Figure S2. The XRD results showed that the optimum peak was obtained in samples with annealing temperature of 1100C. It was found that the degree of crystalline PVDF film was increased by increasing the annealing temperature. The values of the β fraction of PVDF samples for annealing temperatures of 70°C, 80°C, 90°C, 100°C, and 110°C were 37%, 38%, 44%, 50% and 58%, respectively.

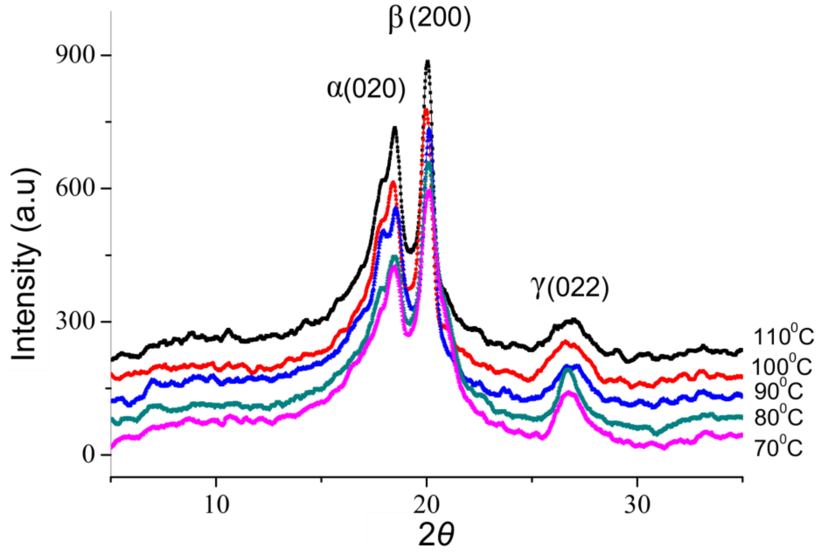

Figure S2. The XRD results for PVDF film with different annealing temperature.
